# Blast-Assisted Subsurface Characterisation Using a Novel Distributed Acoustic Sensing Setup Based on Geometric Phases

**DOI:** 10.3390/s24010030

**Published:** 2023-12-20

**Authors:** Sabahat Shaheen, Konstantin Hicke, Katerina Krebber

**Affiliations:** Bundesanstalt für Materialforschung und -Prüfung (BAM), Unter den Eichen 87, 12205 Berlin, Germany; konstantin.hicke@bam.de (K.H.); katerina.krebber@bam.de (K.K.)

**Keywords:** distributed acoustic sensing, distributed fiber optic sensing, geometric phase, seismology, subsurface characterisation, surface waves, earthquake monitoring

## Abstract

A novel DAS setup based on geometric phases in coherent heterodyne detection is applied for the first time to the characterisation of the Earth’s subsurface. In addition, an optimisation of the proposed setup in terms of its spatial resolution is also presented for the first time. The surface waves are generated by strong blasts of 25 kg of explosives at a dedicated test site. A 10 km dark fiber link in the vicinity of the test site connected to the test setup records the resulting strain signals. The spike-free and low-noise strain data thus obtained minimize post-processing requirements, making the setup a candidate for real-time seismic monitoring. An analysis of the dispersion characteristics of the generated surface waves is performed using a recently reported optimised seismic interferometric technique. Based on the dispersion characteristics, the shear wave velocities of the surface waves as a function of the depth profile of the Earth’s crust are determined using an optimised evolutionary algorithm.

## 1. Introduction

The characterisation of Earth’s near surface has important applications in natural hazard assessment, resource exploration and planning of civil infrastructure. Earthquake monitoring also benefits from it, as the magnitude of vibrations at a given location depends on the near-surface structure and properties. Given its importance, it remains an ever-evolving area of research spanning diverse techniques from digging boreholes to ground-penetrating radar, electromagnetic induction and seismic surface wave analysis [1].

Seismic surface wave analysis is based on the study of seismic waves travelling along the surface of the Earth. It is an established technique for near-surface characterisation, where usually arrays of geophones are used as sensing points to be cross-correlated with a source. The shear wave velocity of these waves at varying depths of the Earth gives information about the density and composition of the respective subsurface layers [1]. Surface waves may emanate from natural processes occurring within Earth’s mantle or acting from outside, including human-made ones, including regular anthropogenic activity. Surface waves may be artificially induced at specific frequencies and amplitudes using special sources like vibrating trucks or blasts. Several studies show subsurface characterisation using dense geophone arrays whose spatial resolution is at minimum of around 50 to 60 m [2,3,4,5,6,7,8]. The limited density of these arrays as well as the installation cost and difficulty may be a challenge, especially in hard-to-reach areas like the ocean bottom or densely populated urban areas [9].

Distributed Acoustic Sensing (DAS) transforms an optical fiber buried in the Earth’s surface into a several-kilometre-long dynamic strain sensor with a high spatial resolution [10,11]. The spatial density of the optical fiber sensor compared with an array of geophones is far superior, making it an ideal candidate for surface wave monitoring. Moreover, the unused links in the optical fiber telecommunication network already deployed over the Earth’s surface, including the ocean bottom [12,13], densely packed urban areas [14,15,16,17,18,19,20] or other specialised terrains [21,22], are readily available for sensing. The permanent deployment of optical fibers also entails the possibility of continuous monitoring. Considering its unique advantages, application of DAS to surface waves and other seismic applications like earthquake monitoring is an area of active research. The aim is to leverage its full potential and overcome associated challenges such as generation of huge amounts of data and signal fading due to characteristics of the optical fiber medium or the measurement technique [9,23,24,25].

Using the principle of Optical Time-Domain Reflectometry (OTDR), a phase shift experienced at a specific location in the optical fiber is determined, which is directly proportional to the strain at this point [10,26]. The measured phase varies with changes in the refractive index, length or wavelength and can be termed a dynamic phase. Interferometric techniques, such as Mach–Zehnder or coherent homo- or heterodyne detection, may be used to quantify the phase shift [10]. In coherent heterodyne detection, a local oscillator (LO) signal is made to interfere with its backscattered counterpart coming from the fiber-under-test (FUT) [26]. This technique offers a high signal-to-noise ratio (SNR) over a long range and is therefore suitable for seismic monitoring. A challenge of this technique is that the traditional method of measuring the phase assumes both the interfering beams as having identical states-of-polarization (SOPs) [10]. This is not the case in the optical fiber medium, where after the first few meters, the SOP of the probe signal diverges from that of the LO [27]. Resulting polarization mismatches cause unwanted signal fades and are traditionally handled with polarization diversity techniques [28,29,30,31,32,33]. The unwanted peaks in the determined phase due to polarization mismatch fading or unwrapping errors can cause false alarms in applications like earthquake and structural health monitoring [34].

Recently, it was reported that a geometric phase exists in the beating of lightwaves as long as the interfering beams have non-identical SOPs. It is measured per beat period and found to be coupled to the dynamic phase [35]. The said geometric phase was detected with a novel DAS setup based on coherent heterodyne detection [36]. Afterwards, a DAS system utilizing a geometric phase instead of the traditionally measured dynamic phase was demonstrated [37]. The new setup, termed $\phi_{g}$-OTDR [37], is immune from polarization mismatch fading as non-identical SOPs of the interfering beams is a condition for its existence, not a hindrance. Moreover, its calculation does not generate unwrapping errors [35,37], which may cause unwanted phase spikes, especially when the signal-to-noise ratio (SNR) is low [38].

Surface wave monitoring usually requires significant data cleaning and averaging to be able to detect surface waves [9,23,24]. We anticipate that using the less noisy and spike-free data from $\phi_{g}$-OTDR may prove advantageous in this regard. Thus, in this work, we use the $\phi_{g}$-OTDR system for surface wave monitoring. As a source for surface waves, we use a series of two test site blasts of 25 kg of explosive material each, buried underground. Relatively few studies exist that use blasts as a source for surface wave interferometry [17,39]; the present study contributes to this body of work. The dispersion characteristics of the surface waves are extracted using an optimisation of the cross-correlation method [40], termed the fast dispersion method. Further analysis of the dispersion characteristics is performed using an optimised evolutionary algorithm that enables one to extract shear wave velocities as a function of Earth’s crust, given an initial model [41].

Finally, a performance optimisation of $\phi_{g}$-OTDR in terms of its spatial resolution is presented here for the first time. The spatial resolution of $\phi_{g}$-OTDR [37] was earlier reported to be lower than that of standard, phase-sensitive OTDR based on coherent detection of the traditionally measured dynamic phase, ϕ-OTDR [10,42]. This is because the numerical method used to compute the geometric phase relies on dividing the beat period into *N* segments and summing over them. Earlier, a single value was computed over each beat period [36,37], but in the present study, we use a moving/sliding summation so that we obtain a value at every spatial point, thereby bringing the spatial resolution of $\phi_{g}$-OTDR equal to that of standard ϕ-OTDR based on coherent heterodyne detection [10,42].

## 2. Experimental Setup

**Figure 1:**
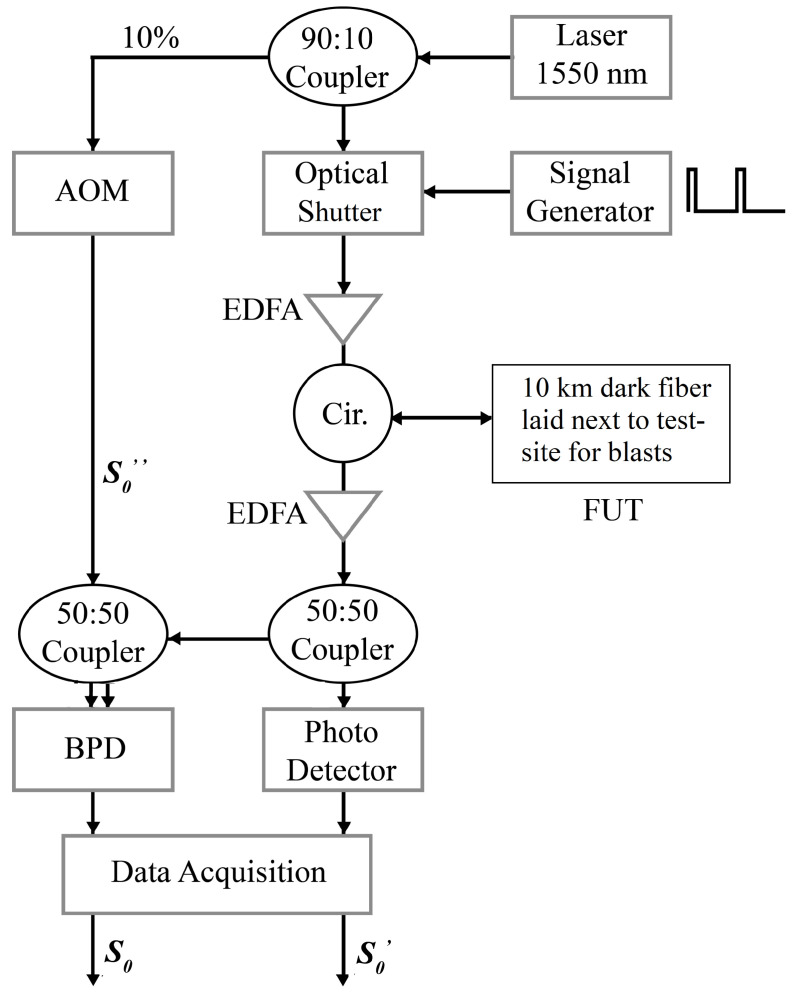
Experimental setup of $\phi_{g}$-OTDR used to interrogate a 10 km dark fiber link surrounding the blast test site. EDFA: erbium-doped fiber amplifier, FUT: fiber-under-test, BPD: balanced photodetector, Cir.: circulator, AOM: acousto-optic modulator.

A DAS setup based on coherent heterodyne detection utilizing a geometric phase instead of the typically measured dynamic phase was recently demonstrated [37]. The same experimental setup is also used in the present study, shown in Figure 1. The calculation of the geometric phase in the beat signal, $S_{0}$, obtained after coherent heterodyne interference of two frequency offset beams of light, $S_{0}^{\prime }$ and $S_{0}^{\prime \prime }$, is accomplished using the following formula [35], where *N* is the number of sections into which a beat period is divided into:

(1) $$\phi_{g}=\pm \pi -\sum_{n=1}^{N}arg[S_{0}^{\prime }exp\left(\frac{-i\pi }{N}\right)+S_{0}^{\prime \prime }exp\left(\frac{i\pi }{N}\right)+2\sqrt{S_{0}^{{}^{\prime }}S_{0}^{\prime \prime }}|\gamma_{0}\left|cos\left(\frac{n2\pi }{N}\right)\right]$$

where $\gamma_{0}$ is calculated as [35]:

(2) $$\gamma_{0}=S_{0}/\sqrt{S_{0}^{{}^{\prime }}S_{0}^{\prime \prime }}$$

The experimental setup consists of a narrow linewidth laser that splits light with a 1550 nm wavelength and 20 mW intensity into probe and local oscillator (LO) branches in a ratio of 90:10, respectively. This splitting ratio [43] works optimally as the LO signal does not need to travel beyond a meter on the optical table and its power easily matches the low backscatter signal arriving from the probe branch. Ratios of 99:1 [26,44] and 50:50 have also been reported [42].

The intensity of the LO, $S_{0}^{\prime \prime }$, remains constant and therefore has to be measured with an optical power meter only once. In the probe branch, a high-speed optical shutter (Booster Optical Amplifier) is used to carve a pulse train that is sent into an FUT, a 10 km dark fiber link, after amplification by an erbium-doped fiber amplifier (EDFA). The pulse width is 50 ns, while the pulse repetition rate is 500 Hz. The Rayleigh backscattered signal (RBS) from the FUT is amplified once again with an EDFA. It is then split equally using a 1×2 50:50 splitter. One half of this signal is detected directly with a single photodetector, giving us $S_{0}^{\prime }$. The other half is coherently heterodyned with the LO signal using a 2×2 50:50 coupler. However, the LO is first given a frequency upshift, Δf= 110 MHz, by passing it through an acousto-optic modulator (AOM). The subsequent interference between the LO and RBS is detected by a balanced photo-detector, giving us the resulting beat signal, $S_{0}$. The frequency offset between the interfering arms, Δf, determines the period of $S_{0}$.

The outputs from both photodetectors are sampled using an analog-to-digital converter at a sampling rate of 500 MSa/s. The setup thus allows for the calculation of $\phi_{g}$ using Equations (1) and (2) by plugging in the values of $S_{0}$, $S_{0}^{\prime }$ and $S_{0}^{\prime \prime }$ [37]. In the present study, we use an AOM with a Δf= 110 MHz, giving us N=9 over two beat periods, as per Equation (3), where two beat periods are considered to obtain an integer value of *N*. The latter is a function of the sampling frequency $f_{s}$ and Δf, calculated as [37]:

(3) $$N=f_{s}/\Delta f$$

The equivalence between the dynamic phase measured per sample and the geometric phase measured per beat period is given as [37]:

(4) $$\phi =-g\cdot \phi_{g}$$

In this work, we present a performance enhancement by taking a moving summation for the calculation of $\phi_{g}$ using Equation (1). The number of samples covered in one gauge length for the $\phi_{g}$-OTDR was reported as $g_{g}=g/N$ [37], where *g* is the number of samples per gauge length for standard ϕ-OTDR [42]. The spatial resolution of $\phi_{g}$-OTDR was lower because $\phi_{g}$ is measured per beat period using Equation (1). However, now, by taking a moving summation, Equation (1), the spatial resolution of $\phi_{g}$-OTDR becomes equal to that of standard ϕ-OTDR [42]. In other words, the number of samples covered in one gauge length for both systems is now equal and equal to *g*. In the present study, this value is g=25.

A pulse repetition frequency of 500 Hz is employed to interrogate a 10 km FUT, implying the maximum detectable signal frequencies up to 250 Hz. The frequencies of interest that surface waves exhibit lie well below this limit, in the order of tens of Hz [1,45]. The proposed pulse repetition frequency is therefore optimal considering the data acquisition and processing load. A computer program written in Python (v. 3.10) for the calculation of geometric phases and further details of the hardware are available in the Supplementary File S1. Further details about the choice of system parameters are discussed in Section 5.

## 3. Experimental Data

**Figure 2:**
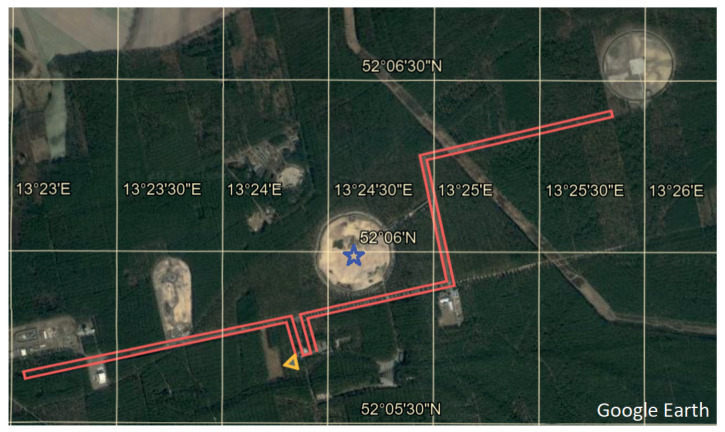
Satellite image of blast test site where the fiber-under-test (FUT) is represented by a line (red), the location of the blasts by a star (blue) and the $\phi_{g}$-OTDR test setup by a triangle (orange) pointing to the beginning of the FUT to which it is connected.

Figure 2 shows a satellite image of the blast test site. The circular sandy patch, marked by a star (blue), is where the explosives were buried underground. The $\phi_{g}$-OTDR setup described above was placed in a server room which is represented by a triangle (orange) pointing to the beginning of the FUT, to which it is connected. The FUT is represented by a line (red), and was laid out in the vicinity of the test site. It is a standard telecommunication FUT which was laid in the late 1990s at a depth of approximately 1.25 m. One can assume it is a loose tube cable with a gel/oil filling without any special armour or rodent protection. It has a diameter of approximately 30 mm and is not further protected by extra pipes. The layout of the FUT comprises an eastern and a western section or loop. The 6 km eastern loop forms a partial L-shape around the test site, with a one-way length of 3 km. The western loop is 4 km long with a one-way length of 2 km. The two sections/loops are connected in the server room to create a 10 km link. The test setup is connected first to the west-side link, which then connects to the longer one on the east-side.

**Figure 3:**
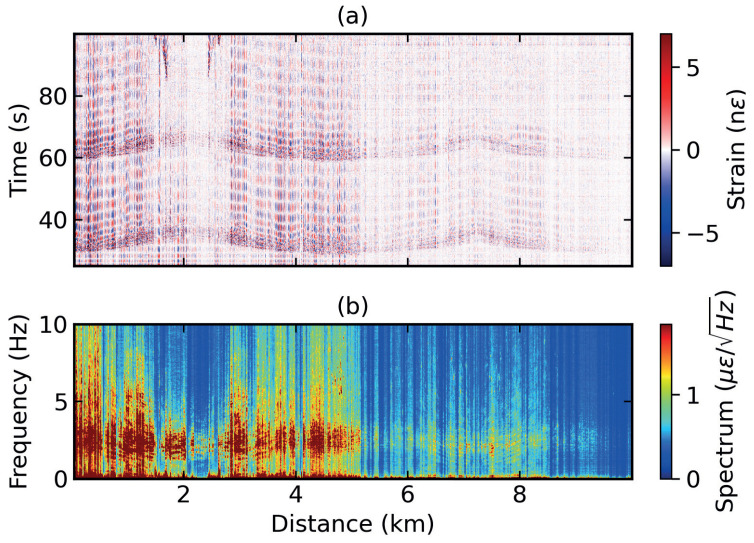
(**a**) Distance–time contour plot of strain data from $\phi_{g}$-OTDR and its (**b**) spectrum.

Figure 3 displays the test site blast signals recorded by the entire 10 km of the optical fiber link. A pair of blasts, utilizing approximately 25 kg of explosive material buried underground, was recorded in a period of 2 min. Figure 3a shows a distance–time contour plot of the resulting strain data at roughly 30 and 60 s. The blast signal was recorded a total of 4 times through the 10 km link. At a fiber distance of around 2.5 km, relatively poor coupling between the fiber cable and the ground is noted. This is due to the cable being buried in a sandy patch around this location. The strain data obtained from $\phi_{g}$-OTDR are a function of both the intensity and the polarisation state of the probe signal with reference to the LO [36]. To remove the dependence of the strain data on intensity, the mean of the time series of strain at each spatial point was subtracted from them and the resulting data were subjected to fifth-order Butterworth high-pass filtering with a cutoff above 0.1 Hz. The frequencies of interest are not anticipated to fall below this range [1,45]; however, the primary function of this filtering is to remove DC components from strain data. However, some traces of change with intensity in the shape of vertical lines along the time-axis can still be seen.

The horizontal bands along the distance axis following the blast signals represent ground vibrations following the blast. Figure 3b represents the spectrum of the strain data obtained through a one-dimensional Fourier transform along the time axis for each measurement position. Most of the power is concentrated in the lower frequency bands between 0 and 5 Hz.

**Figure 4:**
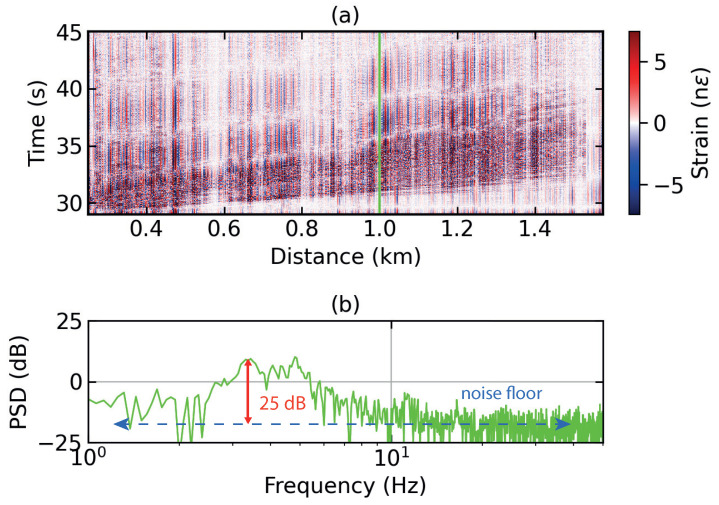
(**a**) Distance–time contour plot of a section of strain data from $\phi_{g}$-OTDR used for dispersion analysis; (**b**) power spectral density (PSD) of a time series of strain at a fiber distance of 1 km (marked by a vertical green line in (**a**)), where the vertical red line indicates the signal-to-noise ratio (SNR) and the horizontal dotted line indicates the noise floor.

To analyse the dispersion characteristics of surface waves, we choose a single blast at 30 s that has been recorded by the first 2 km of the FUT, shown in Figure 4a. This was executed because the signal is the strongest in the beginning of the FUT. The two blasts can be stacked one on top the other in the time domain to enhance the SNR; however, this may mix the surface waves from the two blasts. Alternatively, the four instances of the first blast along the spatial dimension may be stacked. However, such a scheme did not lead to any improvement in the SNR. Moreover, due to the varied coupling conditions that the FUT encounters, the blast signal looks different in the four sections of the fiber. Therefore, we chose only the first instance of the first blast to isolate the surface waves it produces. No improvement is offered by stacking in either the spatial or time domain. Figure 4b shows the power spectral density (PSD) of the time series of strain in nϵ at 1 km, marked by a vertical line (green) in Figure 4a. They are broad-spectrum data distributed along a noise floor of approximately −18 dB with reference to 1 nϵ. An SNR of around 25 dB with respect to the noise floor can be observed at low frequencies between 1.5 and 4 Hz.

## 4. Analysis and Results

### 4.1. Dispersion Image Extraction

As surface waves propagate along the Earth’s surface, the substructure of the Earth causes different frequency components to travel at different speeds, causing dispersion. A common method of calculating a dispersion image involves cross-correlating the time series of strain from all the sensors with respect to a single source, giving us the Green’s function [46] for that source. The sensors then perceive the waves to be emanating from this source (which is accordingly called a virtual source). Cross-correlation is followed by slant stacking [47] to obtain a dispersion image. An optimisation of this method, known as the fast dispersion method, was recently reported [40]. This optimisation is based on isolating repetitive calculations and performing them only once. This implies that the Green’s function of each virtual source is not required to be calculated individually; rather, a parameter termed sigma is calculated once for the whole data-set. The dispersion image for a given virtual source can then be extracted by using the time series of strain at a given spatial location and sigma. We use this method to compute dispersion images, considering its computational efficiency and sharper results [40]. Surface waves are further divided into Rayleigh waves with elliptical motion and Love waves that move horizontally perpendicular to the direction of propagation. The method we follow targets the detection of Rayleigh waves which are slower and last longer compared to Love waves [1,48].

**Figure 5:**
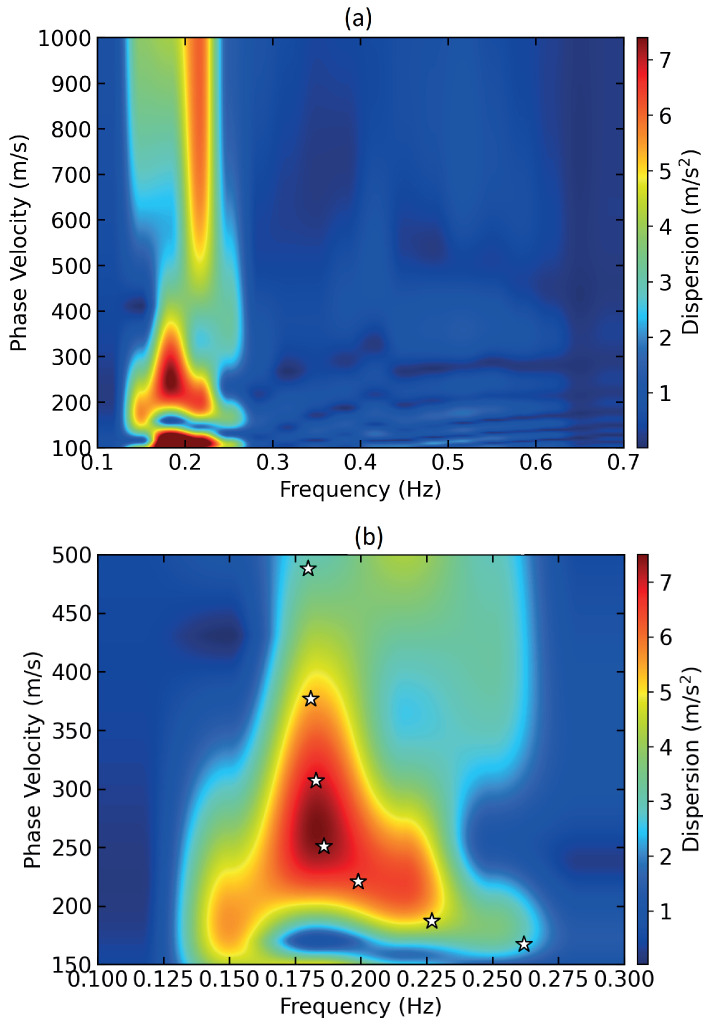
(**a**) Dispersion image; (**b**) fundamental mode of Rayleigh wave where white-colored stars indicate the dispersion curve.

Figure 5a shows the dispersion image of the strain data section shown in Figure 4a, generated by the fast dispersion algorithm described above [40]. The fundamental mode of the Rayleigh surface waves is detected at low frequencies of around 0.2 Hz with the virtual source at a fiber distance of 0.34 km. The chosen virtual source location gives relatively strong dispersion values compared with other points in the 2 km fiber section chosen for this analysis. It may be that the Earth’s near surface changes drastically from one point to the next; however, in our study, the differences in the mode shapes and dispersion values at various points along the chosen fiber section are overall not so drastic. The phase velocity goes up to approximately 490 m/s. A dispersion curve, marked by white stars, is obtained by following the fundamental mode along the phase velocity and frequency axis. The values obtained were interpolated to obtain uniform samples along the frequency axis and processed further for extracting shear wave velocities.

### 4.2. Dispersion Curve Inversion

Dispersion curve inversion is a process in which subsurface layer thicknesses and shear wave velocities, $V_{s}$, of the surface waves for every layer are determined, given a specific dispersion curve. $V_{s}$ is directly related to the shear and elastic modulus of the material and may then be used to determine the material properties of the Earth’s layers [1]. Accurate estimation of subsurface features is challenging, as more than one solution may fit the curve or the algorithm may converge to a local maximum/minimum. Several algorithms have been devised accordingly, including full-waveform inversion and Bayesian inversion using the Markov Chain Monte Carlo algorithm, respectively [45]. Genetic or evolutionary algorithms have also been used, where the principle of ’multiply, vary, let the fittest survive’ is applied to generate an initial set of models, from which the ones that fit best to the dispersion curve survive in successive iterations [41].

For inversion of dispersion curves, we used an evolutionary algorithm known as competitive particle swarm optimisation (CPSO), which is available as an open-source Python package, evodvinv [41,49]. It is based on the evolutionary patterns of swarms, whereas the optimisation offered by CPSO avoids local minima/maxima by adding competition between the models [41]. An initial model of the Earth’s surface, given in Table 1, was used as input to the algorithm. The algorithm serves to refine this model to fit with the frequency versus phase velocity values extracted from the dispersion image. The model consists of four layers with varying thicknesses and shear wave velocity values. A population size of 50 was used with 1000 iterations.

**Figure 6:**
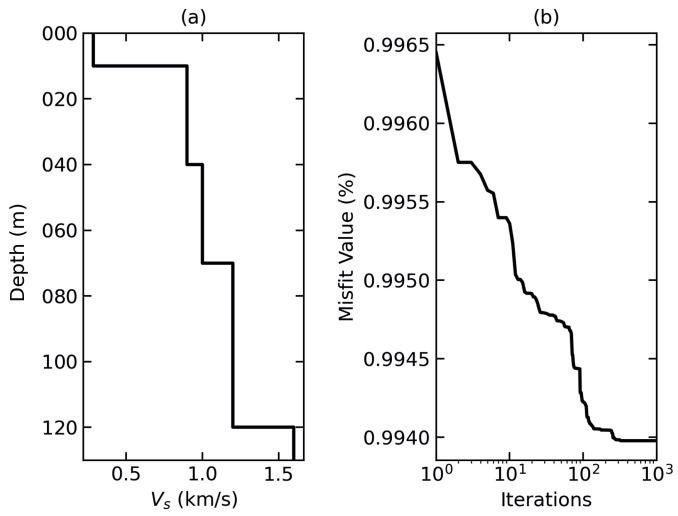
(**a**) Depth vs. shear wave velocity, $V_{s}$, profile obtained from inversion of the dispersion curve, (**b**) misfit values corresponding to (**a**).

Figure 6a shows the results of dispersion curve inversion. Overall, the $V_{s}$ at shallow depths of a few meters is 0.2 km/s, but increases gradually to over 1.5 km/s at the maximum penetrated depth of 130 m. The increase in $V_{s}$ with depth may be attributed to hardening of soil with depth. Figure 6b shows the misfit values, which represent the error in fitting the curve to the model. Misfit values should be as low as possible, with 1% considered low enough [45]. For our results, these values remain close to 1%, showing a close fit.

## 5. Discussion

In the experimental setup described in Section 2, an optical shutter is used for pulse carving, while an AOM is used in the LO only to shift the frequency. An AOM can be used instead of an optical shutter in the probe branch for both pulse carving and frequency shifting. The proposed scheme is chosen due to the very short rise-and-fall time of 1 ns of the optical shutter and the driver circuitry used with it, the details of which are given in the Supplementary File S1. In practice, the rise-and-fall times are limited by the electrical signal generator to 2.9 ns. The high switching speed of the optical shutter enables the setup to effectively reach its theoretical spatial resolution. In the present study, the spatial resolution is 5 m, as a 50 ns pulse train is used for interrogating the FUT. The overall rise-and-fall time of around 6 ns does not significantly lower the theoretical spatial resolution of the system. The minimum gauge length is the same as the spatial resolution of the system, but it can be increased in post-processing, similar to coherent heterodyne-based ϕ-OTDR that uses dynamic phases [42].

The maximum measurement range of the proposed setup has not been determined experimentally; however, we can infer the following: the calculation of geometric phase relies on the measurement of the beat signal, as is the case for ϕ-OTDR based on coherent heterodyne detection. However, in addition, we also need to directly detect the backscatter intensity. Therefore, the range of the proposed system may be theoretically limited by the successful direct detection of backscatter intensity or of the beat signal’s amplitude. Moreover, distributed amplification or the use of repeaters inside the FUT may also influence the measurement range.

The cable installation depth is roughly the same as that of the explosives buried underground. The effect of cable depth relative to the signal source has not been determined. However, it is noted that cable directivity, that may indirectly be related to the cable depth relative to the source, does play a role when DAS is employed for seismic sensing [50]. The effect of directivity is also not explored in the current study. The Rayleigh waves that are to be detected are elliptical and therefore possess both vertical and horizontal dimensions as they propagate across the Earth’s near-surface [1]. Thus, a relatively uniform directivity is assumed across the length of the 2 km fiber section in both vertical and horizontal dimensions.

Finally, the increase in spatial resolution of the system is a theoretical principle whose effects can be seen directly in the strain data shown in Figure 3a and Figure 4a and indirectly in the dispersion images shown in Figure 5. However, the higher spatial resolution is relative to the study presented in [37], where the same setup was tested in a lab environment but with a relatively lower spatial resolution. The system’s immunity to polarisation mismatch fading and phase unwrapping errors was also demonstrated in the latter study. The current work focusses on successful detection of Rayleigh surface waves with minimal data cleaning required due to the absence of spikes and fades that result from the absence of polarisation mismatch fading and unwrapping errors in a $\phi_{g}$-OTDR system.

## 6. Conclusions

We firstly optimise the proposed DAS setup in terms of its spatial resolution based on coherent heterodyne detection utilizing a geometric phase and then demonstrate its feasibility for subsurface characterisation. We perform a dispersion analysis of the surface waves that have been generated by test site blasts and detected by a 10 km dark fiber link using the proposed setup, $\phi_{g}$-OTDR. The fundamental mode of Rayleigh surface waves is detected at a frequency of around 0.2 Hz with phase velocities between 150 and 500 m/s. The shear wave velocities of the Rayleigh surface waves are between 0.2 and 1.5 km/s, gradually increasing with depth and penetrating up to 130 m. The shear wave velocities can be used to determine the material composition of Earth’s layers, thus characterising the subsurface. The strain data from $\phi_{g}$-OTDR are free from polarisation mismatch fades, unwrapping errors and associated spikes, and therefore require minimal post-processing. Real-time seismic monitoring may benefit from the reduced data processing time. The given method can be used for earthquake monitoring and associated disaster response planning, as the speed of waves generated by earthquakes also depends on the subsurface structure.

## Figures and Tables

**Table 1:** Initial model of the Earth’s sub-surface [45].

Layer No.	Min. Thickness (m)	Max. Thickness (m)	Min. Velocity (m/s)	Max. Velocity (m/s)
1	10	60	250	700
2	30	100	300	900
3	30	100	400	1000
4	50	100	600	1200
5	50	100	900	1600

## Data Availability

Data are contained within the article and supplementary materials.

## Supplementary Materials

The following supporting information can be downloaded at https://www.mdpi.com/article/10.3390/s24010030/s1, Supplementary File S1: A python code for calculation of geometric phases and details of the hardware used in the experimental setup.

## Abbreviations

The following abbreviations are used in this manuscript:

AOM	Acousto-optic Modulator
BPD	Balanced Photodetector
Cir.	Circulator
CPSO	Competitive Particle Swarm Optimisation
DAS	Distributed Acoustic Sensing
EDFA	Erbium Doped Fiber Amplifier
FUT	Fiber-under-Test
LO	Local Oscillator
OTDR	Optical Time-Domain Reflectometry
PSD	Power Spectral Density
RBS	Rayleigh Backscatter
SNR	Signal-to-Noise Ratio
SOP	State of Polarization
