# Supplementary material for: Blast-Assisted Subsurface Characterisation Using a Novel Distributed Acoustic Sensing Setup Based on Geometric Phases
Source: Sensors (Basel). 2023 Dec 20;24(1):30. doi: 10.3390/s24010030 (PMC10780498; doi:10.3390/s24010030)
Supplement: Supplementary file 1 [file sensors-24-00030-s001.zip › sensors-2751758-supplementary.pdf]

Blast-Assisted Subsurface Characterisation Using a Novel Distributed Acoustic Sensing Setup Based on Geometric Phases

SUPPLEMENTAL MATERIAL

A Python code to calculate geometric phase is provided. We also compute the traditionally measured dynamic phase for comparison at the end.

H5 file format is used to extract raw data and store processed data. CUDA multi-processing (using graphic card) is used for fast processing.

Models/part numbers of the hardware used in the setup are given at the end of this document.

Sabahat Shaheen, Konstantin Hicke, Katerina Krebber

Bundesanstalt für Materialforschung und -Prüfung (BAM), Unter den Eichen 87, 12205 Berlin,

Germany

sabahat.shaheen@bam.de

```python
# Code created by Sabahat Shaheen

import h5py

import cupy as cp


import matplotlib

import matplotlib.pyplot as plt

from filtering import hilbert as hilbert_transform

# filtering is an equivalent Python package used with cupy

# from scipy.signal import hilbert as hilbert_transform (if not using cupy)


# input file with data. It consists of two data streams coming from the analog- to-digital Convertor (ADC) which converts the output of the two photodetectors to electrical domain. The output of the single photodetector, S0' (in the text of the paper) is represented as So1. While the output from the balanced photodetector is So.

file_name = '20230510_1259.h5'

So2 = 60e-6    # intensity of local oscillator (S0'' in the paper)


# VARIABLES RELATED TO FIBER-UNDER-TEST

# Pulse width generated by the Signal Generator for modulating the output of Laser (50e-9 s for blast or 20e-9 s for traffic scenario)

PulseWidth = 50e-9


# Sampling Rate of the Analog-to-Digital Convertor (ADC), 500 MSa/s at which photodetector outputs are sampled.

SampRate = 500000000
```

Pulse Repetition Frequency provided by Signal Generator (500 Hz for blast or 1000 Hz for traffic)

SigFreq = 500

Samples per Cycle are the samples recorded by the ADC, 2**16 for blast (10 km) and 2 ** 13 (1 km) for traffic (Samples per Cycle depend on fibre length assumed).

SamPerCyc = 2**16

Spatial Resolution of the setup in [Samples/meter], calculated as:

$$\text{Spatial Resolution} = \frac{\text{Sampling Rate [samples/second]}}{\text{Speed of light in fiber/2 [meter/second]}}$$

SpatialRes = cp.ceil(SampRate/1e8)

fiber distance vector

dist = cp.linspace(0, 1e8*(SamPerCyc/SampRate), SamPerCyc, endpoint=True)

Gauge Length [m] =Pulse width [s]*$\frac{Speed\ of\ light\ in\ fiber\ (2e{-}8)\ [m/s]}{2}$

G = PulseWidth*1e8

Number of samples included in one Gauge Length, G

g = int(SpatialRes*G)

VARIABLES FOR CALCULATION OF GEOMETRIC PHASE

```
# N is the number of sections into which a beat period is divided. We consider two beat periods to get an integer value.

# N = 9 for AOM with 110 MHz frequency offset and sampling rate of 500 MSa/s

N = 9

n = cp.asarray(range(1, N))

factor = cp.cos((2*cp.pi*n)/N) # Constant term in in Eq. 1 (used later)

# The data is processed in loops where each loop will process a block of data with 2**24 samples.

block = 2**24


# total samples in the entire data

with h5py.File(file_name, 'r') as f:

    totSamples = len(f["data"])


# number of loops required for the entire data

nloops  = totSamples//block


  # no. of pulses (traces or time-series of strain) in each processing block

nPulses = block//SamPerCyc


# time vector for a given loop

time = cp.linspace(0, (1/SigFreq)*nPulses//2, nPulses//2, endpoint=True)


offset  = 0       # variable used to offset the data in every loop
```

factors to convert from photo-detector signals to Volts

cfac1 = ((2.500/8191)/(1*1.4e3*10)) # single photo-detector

cfac2 = (2.500/8191)/(1*15e3) # balanced photodetector

Create .h5 files to write processed data. We calculate both the dynamic phase and geometric phase and corresponding strain.

```
with h5py.File('processed.h5', 'w') as p:
    p.create_dataset("geo_strain", (0, SamPerCyc-g), chunks=(nPulses//2, SamPerCyc-g), maxshape=(None, SamPerCyc-g))
    p.create_dataset("dyn_strain", (0, SamPerCyc-g), chunks=(nPulses//2, SamPerCyc-g), maxshape=(None, SamPerCyc-g))
```

Start the loop, where strain values for a block of data is calculated per loop from dynamic and geometric phase.

```
    while(nloops>0):
        with h5py.File(file_name, 'r') as f:
```

Read raw data (output of single photodetector, So1 and output of balanced photodetector, So2).

```
            data = cp.asarray(cp.asarray(f['data'][offset:offset+block]))
        So  = data[0::2]*cfac2
        So1 = data[1::2]*cfac1
```

With reference to Eq. 2:

The two beam intensities are multiplied, and their square root is taken. Beat signal’s amplitude is then normalised with it.

```
        arr = cp.sqrt(cp.abs(So1)*So2)
```

However, to avoid divide by zero, the values where So1 is zero are replaced by the first value in the time-series.

```
        arr[arr==0] = arr[0]
```

Beat signal’s amplitude is then normalised with it.

```
gamma_0 = cp.abs(hilbert_transform(So/arr, axis = -1))
```

With reference to Eq. 1, individual terms are calculated:

```
term1 = So1*cp.exp((-1j*cp.pi)/N)

term2 = So2*cp.exp((1j*cp.pi)/N)

term3 = 2*arr*gamma_0
```

The third term require convolution with factor = cp.cos((2*cp.pi*n)/N). First term is also convolved with ones so that the two can be added.

```
term1_conv = cp.convolve(term1, cp.ones((N-1)), 'valid')

term3_conv = cp.convolve(term3, factor, 'valid')
```

Geometric phase is calculated by adding all the terms as per Eq. 1

```
phi_PB = cp.pi - cp.angle(term1_conv + term2 + term3_conv)
```

A few missing samples at the end are replaced by zeros

```
phi_PB = cp.append(phi_PB, cp.zeros((1, N-2)))
```

Geometric phase vector is reshaped into distance-time frame.

```
phi_PB    = cp.reshape(phi_PB, (nPulses//2, SamPerCyc))
```

Differential phase is calculated, which is proportional to strain.

```
dphi_PB   = phi_PB[:, g:] - phi_PB[:, :-g]
```

Geometric phase in nε is finally calculated.

```
strain_PB = ((1550.12e-
9)*dphi_PB/(4*cp.pi*1.4682*G*0.78))*1e9*g
```

The .h5 files are re-sized and the block of geometric phase calculated in this loop is saved in it.

```
p["geo_strain"].resize((p["geo_strain"].shape[0] +
strain_PB.shape[0]), axis = 0)

p["geo_strain"][-strain_PB.shape[0]:] = cp.asnumpy(strain_PB)
```

```
# CALCULATION OF DYNAMIC PHASE

# To compare our results, dynamic phase is also calculated using the
method given in state-of-the-art.

# To calculate dynamic phase, we extract phase from the Hilbert
transform of the beat signal.

        phase  = cp.angle(hilbert_transform(So, axis = -1))

# Dynamic phase vector is reshaped into distance-time frame.

        phase  = cp.reshape(phase, (nPulses//2, SamPerCyc))

# Differential phase is calculated, which is proportional to strain.

        dphase = phase[:,g:] - phase[:,:-g]

# Dynamic phase in nε is finally calculated.

        strain_CH = ((1550.12e-
9)*dphase/(4*cp.pi*1.4682*G*0.78))*1e9

   # The .h5 files are re-sized and the block of dynamic phase
calculated in this loop is saved in it.

        p["dyn_strain"].resize((p["dyn_strain"].shape[0] +
strain_CH.shape[0]), axis = 0)

        p["dyn_strain"][-strain_CH.shape[0]:] = cp.asnumpy(strain_CH)

# the loop number is decremented till we reach a value of zero.

        nloops = nloops – 1

# variable offset is advanced by the block of samples = 2**24

        offset = offset + block

# loop number is printed on screen

        print("strain ... ", nloops)


# …………………………. END OF CODE ……………………………………….
```

Models of the hardware used in the setup:

LASER: Orion RIO ORION Laser Module

- Phase Noise: Grade 3

- Linewidth: ≤ 5 kHz (Lorentzian)

OPTICAL SWITCH: Polarization-Dependent Optical Switch

(BOA 100 4PXS)

Driver: PicoLAS short pulse driver (BFS-VRM 03 HP / LP)

- Rise/fall time 1 ns
-

ACOUSTO-OPTIC MODULATOR

T-M110-0.2C2J-3-F2S with driver (2910 series)

- Frequency offset: 110 MHz

EDFA (Pulsed, used before transmitting pulses into the FUT):

C-band pulsed EDFA by Beogold

-Pulsed output for high SNR

EDFA (used at the receiver)

MAFA 5000 series by emcore (now discontinued)

ADC: M4i.44xx-x8 - 14/16 bit Digitizer up to 500 MS/s by Spectrum Instruments

Single Photo-detector (TTI 525, now discontinued)

Balanced Photodetector: Thorlabs PDB4x5
